# Successful Pulmonary Endarterectomy in a Patient with Klinefelter Syndrome

**DOI:** 10.1155/2012/104195

**Published:** 2012-12-13

**Authors:** E. Wierda, H. J. Reesink, H. Bruining, O. M. van Delden, J. J. Kloek, P. Bresser

**Affiliations:** ^1^Departments of Cardiology, Onze Lieve Vrouwe Gasthuis, 1090 HM Amsterdam, The Netherlands; ^2^Department of Respiratory Medicine, St. Antonius Ziekenhuis, 3430 EM Nieuwegein, The Netherlands; ^3^Department of Pulmonology, Academic Medical Center of the University of Amsterdam, 1105 AZ Amsterdam, The Netherlands; ^4^Department of Respiratory Medicine, Onze Lieve Vrouwe Gasthuis, 1090 HM Amsterdam, The Netherlands; ^5^Department of Psychiatry, Rudolf Magnus Institute of Neuroscience, 3584 CG Utrecht, The Netherlands; ^6^Department of Radiology, Academic Medical Center of the University of Amsterdam, 1105 AZ Amsterdam, The Netherlands; ^7^Department of Cardiothoracic Surgery, Academic Medical Center of the University of Amsterdam, Amsterdam, The Netherlands

## Abstract

Klinefelter syndrome (KS) is a frequent genetic disorder due to one or more supernumerary X chromosomes. KS is associated with an increased risk for venous thromboembolic events like deep venous thrombosis and pulmonary embolism. This paper describes a 37-year-old male patient with KS referred to our tertiary center with chronic thromboembolic pulmonary hypertension, and who was successfully treated by pulmonary endarterectomy.

## 1. Introduction

Klinefelter syndrome (KS; 47, XXY or higher aneuploidies) is a complex genetic disorder with highly variable endocrinological, metabolic, morphological, and neurobehavioral manifestations of altered X-chromosomal gene expression. Recent studies estimate the prevalence of KS 1 : 640 [1, 2], which makes it the most prevalent aneuploidy in males and also the most frequent cause of male infertility. KS is also associated with an increased risk for venous thromboembolic events (VTEs) like deep venous thrombosis (DVT) and pulmonary embolism (PE) [3]. Although the underlying mechanism is incompletely understood, it is thought to be related to a hypogonadism syndrome leading to an increased synthesis and activity of plasminogen activator inhibitor-1 (PAI-1) and thus a reduced fibrinolytic activity [3]. It might be hypothesized that patients with KS are also at higher risk to develop chronic thromboembolism and chronic thromboembolic pulmonary hypertension (CTEPH) [4]; however, no such case was reported before. Here, we report a case of a 37-year-old KS patient who suffered from CTEPH, and who was successfully treated by pulmonary endarterectomy.

## 2. Case Report

A 37-year-old male was referred to the CTEPH center of the Academic Medical Center of the University of Amsterdam for the analysis of suspected CTEPH. At the age of 30, he was diagnosed with KS by genetic counseling (karyotype 47, XXY). One year later, following a high energetic trauma complicated with osteomyelitis of the right femur, he presented with acute onset dyspnea. By computed tomographic (CT) pulmonary angiography, acute bilateral PE was diagnosed as sequelae of a DVT of the right leg. The patient's family history was negative for VTE. Anticoagulant treatment was instituted with vitamin K antagonists for total duration of six months, whereupon he recovered promptly. 

**Figure 1:**
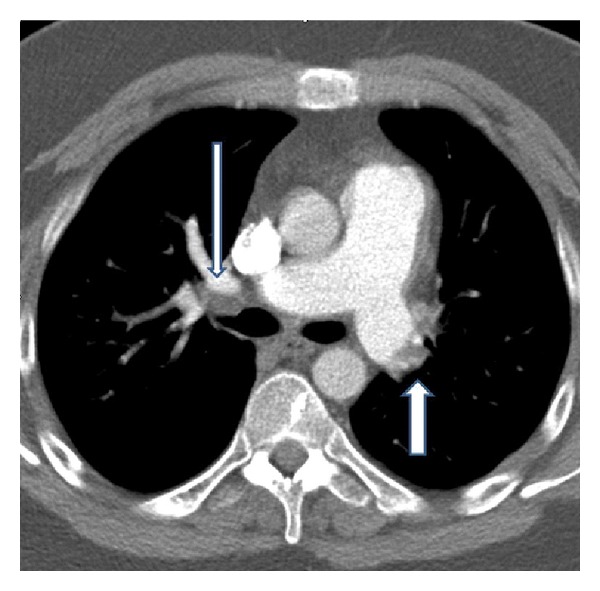
Computed tomography angiogram showing chronic thromboembolic clots in the central left and right pulmonary arteries (arrows).

**Figure 2:**
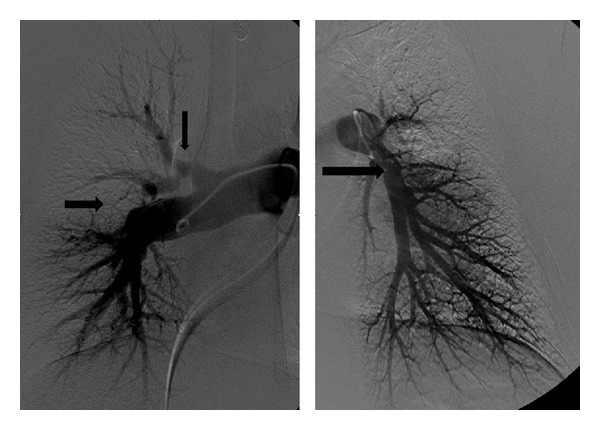
Distraction pulmonary angiogram of the right and left pulmonary artery demonstrating a web and acute stops in the right upper and lower lobe arteries as well as in the left lower lobe artery (arrows).

Six years later, however, he started to suffer from slow onset dyspnea on exertion. Perfusion scintigraphy showed multiple segmental and subsegmental defects, consistent with possible pulmonary embolism. Since anticoagulant treatment for six months did not improve his complaints, he was referred to our hospital. At referral, the patient was in no respiratory distress at rest, with a peripheral oxygen saturation of 99%. He was mildly retarded and obese (body mass index 31.7), had a thickened neck, and a widened forehead with little hair growth. Systemic blood pressure was 120/80 mmHg. Cardiac and pulmonary examinations were normal, except for a split second heart tone. No peripheral oedema was observed. Laboratory tests were within normal range; NT pro-BNP:120 micrograms/mL (*N* < 200 pmol/l, [5]). No coagulation abnormalities were detected, except for Factor VIII which was slightly elevated 211% (*N* < 150%). CT angiography demonstrated large, organized thrombi in the left main pulmonary artery, as well as in the right upper lobe multiple webs (Figure 1). Pulmonary angiography confirmed the diagnosis of proximal CTEPH with multiple webs on both sides and a central pouch in the left main pulmonary artery with diminished perfusion to the left upper lobe (Figure 2). Exercise capacity was decreased; the distance walked in the 6-minute walk test (6-MWD) was 480 meters (predicted value of 658 meters [6]). Echocardiography showed a dilated and hypertrophied right ventricle; systolic right ventricular function was normal (TAPSE 2.4 cm). Estimated systolic pulmonary artery pressure (SPAP) was 65 mmHg (*N* < 40 mmHg). Left ventricular dimensions and function were normal. Right heart catheterisation demonstrated a pulmonary artery pressure of 59/29 mmHg, mean PAP of 43 mmHg (*N* < 25 mmHg), cardiac output of 6.0 L/min, pulmonary wedge pressure of 6 mmHg, mean right atrial pressure of 11 mmHg, and the calculated pulmonary vascular resistance (PVR) of 493 dynes·s·cm^−5^.

**Figure 3:**
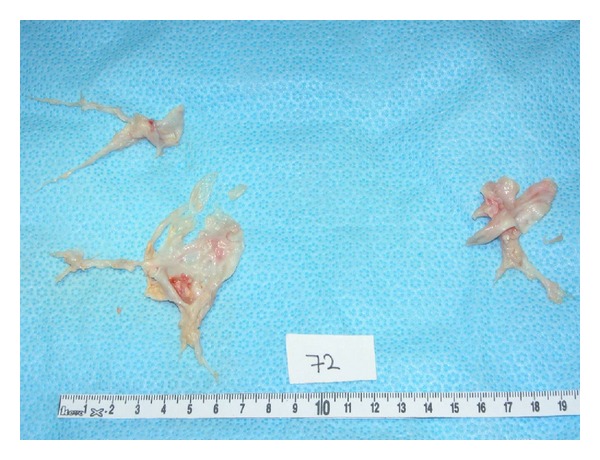
Chronic thromboembolic material obtained by pulmonary endarterectomy demonstrating central pouches from the right and left main pulmonary artery.

The patient was diagnosed with proximal CTEPH. His functional impairment was classified as New York Heart Association (NYHA) III/IV; that is marked limitation in activity due to symptoms, even during less-than-ordinary activity. Without treatment, he had an estimated 5-year survival of less than 30% [7]. A multidisciplinary team consisting of a pulmonologist, a radiologist, and a thoracic surgeon considered the patient eligible for pulmonary endarterectomy (PEA). A PEA was performed, as previously described, under deep hypothermia and cardiac arrest [8]. The organized thrombi were successfully removed (Figure 3). Two days after surgery, mean PAP was 22 mmHg. The patient recovered promptly without any complication and could be dismissed after 2 weeks. At 1-year followup, the patient was in NYHA functional class I/IV (no symptoms, and no limitation in ordinary physical activity); subjectively, his exercise tolerance had fully normalised, and the 6-MWD had increased to 580 meters. Echocardiography at one year after surgery showed a normalized diameter of the right ventricle and an estimated systolic PAP of 27 mmHg. At 5-year followup, now, the patient is still in NYHA functional class I/IV, and he walked 630 meters in the 6-MWT.

## 3. Discussion

KS is associated with substantial morbidity [9] and increased mortality [10] with an increased relative risk of death due to diabetes, cardiovascular, respiratory, and gastrointestinal disorders [11]. KS is caused by chromosomal aneuploidy, in 80% of cases due to chromosome aberration 47 XXY [12]. The prototypic KS man has traditionally been described as tall, with small testes, and decreased verbal intelligence, but the clinical picture may range variably [9]. Here, we reported a patient with KS diagnosed at the age of 30, who developed symptomatic CTEPH six years after an acute pulmonary embolism. 

CTEPH results from incomplete resolution of the vascular obstruction caused by pulmonary thromboembolism [13]. The incidence of CTEPH after acute pulmonary embolism is unknown but may be as high as 4% in patients after a first acute pulmonary embolism [13]. If left untreated, prognosis is poor and survival is related to the degree of pulmonary hypertension. Five-year survival in patients with a mean PAP above 30 mmHg is 30%, whereas patients with a mean PAP above 50 mmHg have a 5-year survival of only 10% [7]. Although specific pulmonary antihypertensive medication is currently available (such as endothelin-1 antagonists, phosfodiesterase-5 inhibitors, and prostanoids [14, 15]), PEA represents the therapy of choice for patients with surgically accessible thrombi [8, 16–18]. After surgery, most patients experience a substantial hemodynamic and functional improvement and have an excellent long-term survival [8, 16, 17, 19].

CTEPH manifested in this patient six years after the documented acute pulmonary embolism. Moreover, he had been fully asymptomatic for several years. Therefore, it is highly unlikely that it has been merely the consequence of this episode. Pengo et al. showed that CTEPH manifests itself within 2 years after an episode of pulmonary embolism [13]. Retrospectively, he did not recall any other acute episode. So, whether the development of CTEPH in our patient was caused by recurrent VTE, in situ thrombosis, or both remains unknown. 

Thromboembolic disease is frequently observed in KS [3, 12]; however, the increased incidence of VTE is incompletely understood yet. The hypogonadism syndrome leads to an increased synthesis and activity of PAI-1 [20]. Plasma levels of PAI-1 are inversely correlated to the testosterone levels, and positively to the extent of obesity, as expressed by the BMI [21]. However, it is believed by most authors that additional thrombophilic conditions, such as Protein C deficiency [22], Factor V Leiden, or Factor II mutation, are mandatory to cause severe VTE [3, 23, 24]. Obesity that is also frequently observed in KS patients may serve as an additional independent risk factor for VTE in these patients [25]. In the present case, osteomyelitis in the absence of hormonal substitutional therapy may have triggered the initial DVT. Also, at referral to our hospital, Factor VIII levels were mildly elevated. Factor VIII is a well-recognised risk factor for single [26] and recurrent VTE [27]. Moreover, elevated levels of Factor VIII have been described in CTEPH patients [28].

As in VTE, elevation of PAI-1 activity is also considered to play a role in the pathogenesis of (postthrombotic) leg ulceration observed in KS patients [29, 30]. Postthrombotic venous ulceration is observed in up to 13% of KS patients [31]; in fact, this may trigger suspicion on KS [32]. In addition, the frequency of pulmonary embolism in KS is likely to be 5–17 times higher [3], and also a higher incidence of chronic thromboembolism in KS may be expected. This is important as most men with KS (64%) remain undiagnosed due to the highly variable and heterogeneous clinical presentation and insufficient professional awareness of this highly frequent syndrome [33]. Early diagnosis of KS might reduce the risk for VTE and timely androgen treatment will exert a favorable profibrinolytic effect [12]. Thus, both from the viewpoint of early detection as well as for early intervention, the association of KS with thromboembolic events needs to be more firmly acknowledged. 

In conclusion, future studies are warranted to unravel the pathogenesis of VTE and the incidence of chronic thromboembolism in KS patients. However, given the increased incidence of VTE, in KS patients presenting with dyspnoea with or without a previous history of VTE, CTEPH must be considered. CTEPH is a life-threatening yet potentially curable form of pulmonary hypertension if diagnosed early in the course of the disease [16].

## Abbreviations

KS: Klinefelter syndrome
VTEs: Venous thromboembolic events
DVT: Deep venous thrombosis
PE: Pulmonary embolism
CTEPH: Chronic thromboembolic pulmonary hypertension (CTEPH)
CT: Computed tomographic
NYHA: New York Heart Association
PEA: Pulmonary endarterectomy
PVR: Pulmonary vascular resistance
6-MWT: 6-minute walking test.
